# The effects of demand-resource relationship on work-family conflict under Chinese culture: a cross-sectional study

**DOI:** 10.3389/fpsyg.2024.1334538

**Published:** 2024-05-02

**Authors:** Tianjiao Li, Zhiwei Helian, Ling Hu, Mengyuan Ma

**Affiliations:** ^1^School of Economics and Management, Yanshan University, Qinhuangdao, China; ^2^School of Foreign Languages, Yanshan University, Qinhuangdao, China

**Keywords:** work-family conflict, work demand-family resource, work resource-family demand, dynamic relationship, polynomial regression and response surface analysis

## Abstract

Chinese work and lifestyle are undergoing dramatic changes caused by constantly changing technology and new policies. The demand-resource dynamic relationship, which leads to work-family conflict (WFC), has become increasingly complicated. However, very little is known about the combined effects of different factors from work and family spheres on WFC. This study aims to explore (1) the discrepancy between fit and misfit, (2) the discrepancy of the different degrees of fit, and (3) the discrepancy of the different degrees of misfit from two perspectives: work demand-family resource and work resource-family demand. Data were collected from 745 individuals in China and analyzed using polynomial regression and response surface. The results demonstrate that individuals having low work demand–high family resources experience the lowest WFC, and the fit between work demands and family resources impacts the conflict in a U way. Similarly, high work resource–low family demand results in the lowest WFC; however, the fit between work resources and family demands has negative effects on the conflict. This study took factors from both family and work domains into consideration and explored the effect of their interaction on WFC. By examining the dynamic relationship between demands and resources, adjustments can be made in both domains simultaneously, providing more flexible guidance for management practices that reduce WFC.

## Introduction

Work and family are two of the major themes in adult life. The roles expected of individuals at work and at family are not always compatible, thus leading to conflicts between an individual's work and family life. The issue of work-family conflict (WFC) has always been an interesting topic as it is normally and consistently related to negative outcomes on one's mentality, physiology, and behavior. These outcomes may include stress, sub-health status, sudden death or untimely death, low work efficiency, low job satisfaction and low family satisfaction (Edwards and Rothbard, 2000; Anderson et al., 2002; Carlson et al., 2011; Žnidaršič and Bernik, 2021). As a result of information technology and new policies in China, the work and family domains have undergone dramatic changes over the last few years. For instance, information technology has facilitated the growth of social media and e-commerce platforms (WeChat, Weibo, Taobao, and Pinduoduo), which have obscured the boundaries between work and family. Social media, similarly, has improved the ease and speed of work demands and their delivery. Technology has objectively increased the time and the intensity of work for individuals. This has implicitly increased the organizational expectations of greater work intensity from its employees, consequently increasing the employee's perceived workload. E-commerce platforms offer the opportunity to work from home. For example, an online store could be opened in Taobao or Pinduoduo. Working from home through e-commerce platforms creates a blurring of work and family roles, which leads to confusion between work time and family time, making it difficult to allocate time effectively and handle important family matters. The recent implementation of the two-child policy imposes a dramatic change in the Chinese family structure. Families are faced not only with the challenge of giving birth to a second child but also with the problems of raising it. This involves a doubling of manpower, energy, financial resources, and other aspects, thus further increasing the pressure on the family. All these changes lead to new work and lifestyles that bring new perspectives to the study of WFC. Therefore, new strategies to reduce WFC are urgently needed.

Previous research on antecedent variables of WFC has focused on work domains that comprise work demands and work resources (Furtado et al., 2016; French et al., 2018). Work demands refer to an individual's ongoing physical and psychological commitment to work (Ten Brummelhuis and Bakker, 2012). Relevant studies have shown that work demands are positively associated with WFC (Li and Jin, 2015; Tement and Korunka, 2015). Work resources mainly consist of the nature of work (autonomy) and organizational support (from colleagues or supervisors), which can mitigate WFC by providing tangible and intangible help (Francis, 2016). While there has been a wealth of research, many studies have focused primarily on work unilaterally, overlooking the fact that family is one of the important domains for individuals, constantly influencing the work domain. Family demands refer to an individual's ongoing physiological and psychological investment in their family. Previous research has indicated a positive correlation between family demands, like age and the number of carers, and WFC (Ten Brummelhuis et al., 2012). Family resources are considered to be emotional and instrumental support from family members. Research has shown that support from family can effectively reduce an individual's experience of WFC (Kirrane and Buckley, 2004). Cohen (1985) proposed the buffering model, which demonstrates that sufficient family support can result in low WFC even when work demands are high. Voydanoff (2005) has also pointed out that WFC emerges from the extent to which work or family resources meet family or work demands. These studies establish that WFC is determined by the interaction of factors from both work and family instead of a single field. Studies on the combined and interactive effects of factors from both domains could have different outcomes. Thus, combining the demands and resources from work and family domains is a new viewpoint to analyze the formation of WFC, which can provide new strategies to mitigate WFC. The combined and interactive effects of factors from both work and family domains can be analyzed from two perspectives—work demand-family resource and work resource-family demand.

Unlike in Western countries, people in China always maintain a tradition of “job priority” (Zhang et al., 2011). China is a collectivist society, and work is considered a means to support and improve one's family. This collectivist idea prompts citizens to work for the welfare of the whole family. Embracing this ethic, working overtime is considered self-sacrifice for the sake of the family; working diligently is a symbol of being responsible (Aryee et al., 1999; Yang et al., 2000). Confucian culture encourages individuals to gain higher social status, which stimulates individuals to work hard to get promotions. Further, social norms also encourage individuals to sacrifice family interests to meet the demand of a bigger collectivism (work or the whole society). This behavior of sacrificing family to meet work demands is normal and even praised in China, while family members are inclined to support and understand work demands. Thus, the resources and demands of work and family have a more prominent combined effect on WFC in the Chinese culture.

This study aims to extend the research on WFC by focusing on the entire interactive process of demands and resources (mis)fit based on the P-E theory (Pervin, 1989) and examines from two perspectives (work demand-family resource and work resource-family demand): (1) the discrepancy between fit and misfit and which can lead to less WFC; (2) the discrepancy of high and low demand-resource fit; and (3) the discrepancy of two kinds of misfit (high resource-low demand and high demand-low resource). We conclude by giving specific and actionable suggestions from four aspects to employers and employees. We contribute to research in this field on several aspects: (1) instead of focusing on single factors, we examined the interaction between factors drawn from both work and family domains; (2) instead of solely focusing on the fit or misfit, we examined two conditions of misfit: high demand-low resource and high resource-low demand, as well as the entire dynamic process of misfit-fit-misfit; and (3) we used polynomial regression and response surface analysis to examine the hypotheses which is more comprehensive, accurate and concept clarified.

## Theoretical foundation and hypotheses building

### P-E fit theory

We developed this study based on the Person-Environment (P-E) fit theory. P-E fit theory is conceptualized as the degree to which a person and environment match or are congruent. This theory was applied broadly in organization management but insufficiently in the work-family field. Based on the connotation of fit, Kristof (1996) illustrated the person-organization fit, needs-supplies fit, and demands-abilities fit, which is also known as the three-factor model of fit. This model infers that there is a huge difference between the skills and demands of a person and the demands and rewards of the job, while the congruence between an individual's characteristics and a work's characteristics can pose a positive influence on the individual's happiness. Applying the person-environment fit theory to work-family interface research reflects the interaction of individual, work, family, and organization and integrates the influences of all these factors on work-family relationships.

Even though the fit of the work-family interface gives us a comprehensive and in-depth understanding of the work-family relationship, studies in this field are still insufficient. Pittman (1994) defined work-family fit as the congruence between work demands and family abilities that can meet those demands, as well as the congruence between family needs or goals and job returns. Voydanoff (2005) defined work-family fit as an internal congruence from the perspective of demands and resources based on the theory of P-E fit. He divided work-family fit into two dimensions: work demands-family resources fit, and family demands-work resources fit. According to his research, fit occurs when family or work resources meet work or family demands, and the more resource exceeds demand, the more fit occurs. However, this kind of definition has the defects of vague theoretical concepts and low reliability of difference. This study defines fit as the equalization of demands and resources, and misfit happens when resources exceed demands or when demands exceed resources.

### Job demand-resource model

Demerouti et al. (2001) proposed the Job Demand-Resource Model (JD-R model) and discussed the relationship between job demands (resources) and job burnout. Based on this model, scholars have gradually extended the application scope of the JD-R model into the field of work-family issues. Previous studies have found that job demands are significantly positively related to WFC (Grzywacz and Marks, 2000; Frone, 2003), and job resources are negatively related to WFC (Grzywacz and Marks, 2000; Maume and Houston, 2001). Voydanoff (2004) demonstrated that job demands have a significant positive relationship with the perceived WFC by hindering the performance of family roles and consuming family resources. He stated that work resources are negatively related to WFC because their skills, material, and spiritual resources can help individuals perform family roles. Different from previous studies, we focus on the dynamic process of work demands and resources fitting with family resources and demands and how it impacts WFC.

### Demands and resources

According to Voydanoff (2004), work and family demands are characteristics of one domain that are associated with processes that limit the ability of individuals to meet obligations in other domains. They include time-based and strain-based demands. Time-based demands in one domain reduce the time or involvement available for participation in another domain, such as working overtime, paid work hours, taking care of children, and housework. Strain-based work and family demands operate as a type of psychological spillover where the strain in one domain is carried over to another that creates strain in the latter, thereby hindering role performance in that domain, such as role overload, job insecurity, role conflict, and kin demand.

In this study, we consider work resources as those that can meet the demands in the family domain, such as work control, social support, and boundary flexibility, as well as support resources, which include policies and programs made by employers to help employees coordinate their work and family responsibilities. Examples are flexible work schedules, household services, and parental leave. Family resources, especially from a spouse or a partner, parents, and children, are those that can meet the time-based and strain-based demands in the work domain, including emotional resources (e.g., listening, feedback) and instrumental boundary flexibility supported resources such as dependent care and household work provided by spouses or other family members.

### Work demand-family resource and WFC

High work demand leads to high WFC as it requires individuals to devote more resources to work, leaving fewer resources to devote to family (Bakker et al., 2008) or even consuming family resources. Family-based work support mostly occurs when spouses or other family members can share housework or take care of dependents. Such support is expected to enhance role performance in both fields by adjusting family duties to accommodate work duties. Even though individuals might confront work overload, spouses or parents sharing housework and taking care of children will lower their family responsibilities to an extent and allow them to devote themselves to work. Thus, low WFC will be perceived. Family resources can help individuals coordinate work and family responsibilities effectively. Thus, the more family resources they embrace, the more they can balance and coordinate work and family life and perceive low WFC.

Ma and Xu (2015) also demonstrated the same results but just focused on fit and misfit and was criticized due to vague concepts. This study argues that there are three relationships between demand and resource—demand exceeds resource, demand equals resource, and resource exceeds demand. We define fit as demand equals resource, and misfit demand exceeds resource, or resource exceeds demand. As illustrated above, the more the family resource exceeds work demand, the less WFC the individual will experience. In short, the fit of the paired factors will not lead to the lowest WFC, but the low-high misfit is the best condition, which leads to the lowest WFC.

H1: Fit between work demand and family resources is not the point at which individuals experience the lowest WFC; WFC will increase as work demand increases toward family resources and continue to increase as work demand exceeds family resources.

While discussing the fit between work demand and family resources, it is essential to be clear that there is a high-level fit and low-level fit between work demand and family resources. For example, a sales manager needs to work overtime (high work demand) while his/her spouse does not work or has a part-time job so that the spouse is able to take care of family issues (high family resources). Although high work demand will increase perceived WFC, high family resources can help the manager to have enough time and energy to work and perceive low WFC. For an employee who does not need to work overtime (low work demand) with a busy spouse who has no time to take care of the family (low family resource), although the lower family resource cannot offer enough help to the employee, low work demand allows that individual ability to adjust time and energy to fulfill family duties. As a result, low WFC will be perceived. Many scholars have extended the JD-R model to work-family studies, which demonstrate that work demand positively affects WFC (Voydanoff, 2004; Zhao and Sun, 2013; Tement and Korunka, 2015). In short, work demand directly impacts WFC, but family resource plays a remedial role in reducing the negative effects of work demand. Work demand dominates the effect process. Further, high work demand is more likely to consume more family resources, and according to the conservation of resources (COR) theory, individuals with low resources are more vulnerable (Hobfoll, 1989).

H2: Individuals who experience high work demand-high family resource fit will perceive high WFC compared to those who experience low work demand-low family resources fit.

In most cases, work demands and family resources are in a state of misfit. The misfit has two directions. First, when work demands are greater than family resources, namely, the misfit between high work demands and low family resources. For example, an employee needs to be expatriated for a long time (high demands), and in the meantime, the spouse cannot take care of the family (low resources). In this situation, the individual will experience high WFC. Second, when work demands are less than family resources, hence a misfit between low work demands and high family resources. In this condition, the work demand is low, and the family has sufficient resources to meet work demands, so the individual experiences extremely low WFC. When resources exceed demand, low work demands do not pose threats to an individual's sufficient resources, and the individual experiences low WFC. On the contrary, in high demand-low resource conditions, high demands require individuals to devote more time and energy, and family resources are insufficient to accommodate work demands, so the individual will perceive high WFC. These two kinds of misfit have totally different effects; thus, instead of solely considering fit or misfit, we examine the discrepancy of misfit. We define high work demand–low family resource as an “inferior” misfit and low work demand-high family resource as a “superior” misfit. Hence, the third hypothesis:

H3: Individuals who experience high work demand-low family resources (“inferior” misfit) will perceive higher WFC compared with those who experience low work demand-high family resources (“superior” misfit).

### Work resource-family demand and WFC

Work resources are negatively related to WFC. Family-supported organizational policies or family-supported supervision can reduce time demands and increase work flexibility (O'Driscoll et al., 2003); thus, employees are more likely to accommodate family demands. Material resources from work can support the daily expenditure of the family, meaningful or decent work makes family members feel proud and esteemed, and work autonomy provides employees with more flexibility and authority, all of which allow employees to coordinate the demands between work and family smoothly (Golden et al., 2006). Also, resources in the work field can help individuals gain access to resources in the family domain. For example, decent jobs give employees a chance to get more support and understanding from family members because they are more willing to share the employees' family responsibilities. Work resources are the buffer for family demand and can counteract the negative impacts of family demand. Therefore, more work resources make it more possible to offset the pressure from family demands and lower perceived WFC.

H4: The fit between work resource and family demand is not the best point at which an individual experiences the lowest WFC; WFC decreases as work resource increases toward family demand and continues to decrease as work resource exceeds family demand.

Accordingly, there is also a high-high fit and a low-low fit between work resources and family demand. High salaries (high work resources) can support high household expenses (higher family demand). Although the family has high demands, sufficient work resources are possible to overcome obstacles and solve problems in the family field. As for low work resources (low work autonomy), even though it can meet low family demands, this fit is a kind of “compromised” fit. According to COR theory, resources have a spiral loss effect, which means individuals with insufficient resources are more vulnerable to the pressure of resource loss. The pressure will further reduce resources and accelerate the loss of resources (Hobfoll, 2011). Individuals with low work resources are more likely to deplete their work resources when they consume them to meet family demands. Most individuals cannot fully meet their family demands. Chinese usually sacrifice family demands to devote themselves to work to gain more resources to support family demands. Thus, work resources dominate the impact of the fitting process.

H5: Individuals who experience low work resource-low family demand fit will perceive higher WFC compared with those who experience high work resource-high family demand fit.

There are two directions in this type of misfit: (1) Family demand exceeds work resources, that is, the misfit between low work resources and high family demands. For example, when work lacks flexibility and autonomy (low work resources), combined with long-term young children care (high family demands), and an individual cannot obtain sufficient resources from work to meet the family demands, the individual will perceive high WFC. (2) Work resource exceeds family demand. Under this condition, sufficient work resources can meet family demands. Thus, individuals will perceive extremely low WFC. Similarly, we consider work resources exceeding family demand as a “superior” misfit and family demand exceeding work resources as an “inferior” misfit. Different from previous studies, this study believes that resources are a strategy to overcome the negative effects derived from demand, and the more resources the individual obtains, the better the possibility of meeting demands.

H6: Individuals who experience low work resource-high family demand misfit (“inferior” misfit) will perceive high WFC compared with those who experience high work resource-low family demand misfit (“superior” misfit).

## Methods

### Research context and sample

In this study, a convenience sampling strategy was adopted. A questionnaire survey was conducted among employed individuals from multiple industries, regions, age groups, and educational backgrounds. At the same time, to compensate for the disadvantages of sample bias and underrepresentation that exist in convenience sampling strategies, data was collected from multiple institutions and enterprises to reduce bias.

The cross-sectional study was conducted from September 17, 2018, to December 19, 2018. We collected our data from multiple organizations in Tianjin, Beijing, Shanghai, Shijiazhuang, Qinhuangdao, and other regions. The local government helped us connect with local institutions and enterprises. Data collection was performed in two ways: (1) By email, mainly through institutions/enterprises contact person who sent the link to the electronic version of the questionnaire to the respondents. The purpose of the survey and the voluntary nature of participation were explained at the beginning of the questionnaire, which contained an electronic informed consent form and could be submitted only if all questions had been answered. Therefore, questionnaire submission was deemed to constitute consent for study participation. (2) On-the-spot recycling, where members of the research group visited the institutions/enterprises, distributed questionnaires to the respondents and collected them on the spot. Respondents were included after signing the informed consent form and could quit completing the questionnaire at any time if they felt uncomfortable.

A total of 800 questionnaires were distributed, and 786 questionnaires were recovered. Excluding invalid questionnaires with obvious patterns of responses and short answer time, the total number of valid questionnaires recovered was 745. The 745 respondents surveyed were from a variety of industries with diverse educational backgrounds (23.6% held a master's degree and above, 53.4% held a bachelor's degree, and 23% held a college degree and below). Among them, 55.6% were male, 73.8% were married, and most respondents had children (53.8%). They were stratified by age into groups aged 29 and below (36.6%), aged 30–39 (48.6%), and aged 40 and above (14.8%). All were older than 18 years. The specific industries the participants were from are (1) Internet companies, (2) transportation, post, and telecommunications, (3) accommodation and catering, (4) finance, insurance, and information consulting, (5) tourism, (6) resident services and medical services, (7) real estate, (8) education, culture, and sports, and (9) others. While the length of their service was different−1–5 years (35.6%), 6–15 years (41.8%), and over 15 years (22.6%)—their position level also varied: general employees (59.3%), junior managers (26.2%) middle managers (12.6%) senior managers (1.9%). It can be seen that the samples have a wide distribution and meet the basic requirements of the study. It is noteworthy that data collection was made before the COVID-19 pandemic.

This study used indirect subjective measurements to measure fit. Participants were asked to rate the perceptive level of each factor. The measurement scales were translated into Chinese according to the “translation/back-translation” procedures by professionals. Before the formal survey, a small sample of MBA students were asked to finish the survey to test the items. Due to cultural differences, some items' expressions were amended for better understanding.

### Measures

#### Work demand and family resource

Work demand was measured using Karasek's (1979) 6-item scale. A sample item was “My job requires me to work for a long time (or work overtime).” Respondents were asked to indicate to what extent they agreed with the item using a 5-point scale (1 = strongly disagree, 5 = strongly agree). A higher score represents higher job demand. The coefficient α for this scale was 0.82. Referring to the work demand measurement items, family resources were measured by requiring the respondents to answer questions such as “My family members can give me support from time, mental, and behavior aspects to deal with my overtime work.” The response options ranged from 1 (strongly disagree) to 5 (strongly agree). A higher score represents higher family resources. The coefficient α for this scale was 0.90.

#### Work resources and family demand

Family demand was measured by Choi and Chen (2006) 4-item scale from the aspects of time, energy, and role stress. A sample item was “Family responsibilities cost me a lot of time.” Respondents were asked to indicate to what extent they agreed with the item using a 5-point scale (1 = strongly disagree, 5 = strongly agree). A higher score represents higher family demand. The coefficient α for this scale was 0.90. Referring to the family demand's measurement items, work resource was measured by requiring the respondents to answer questions such as “My work can give me support from material and spiritual aspects to deal with the problem of spending much time on family responsibilities” (1 = strongly disagree, 5 = strongly agree). A higher score represents higher work resources. The coefficient α for this scale was 0.74.

#### Work-family conflict

Work and family conflict was measured by a 4-item scale describing WFC (Wayne et al., 2004). A sample item of WFC was “Your job reduces the effort you can give to activities at home.” Participants were asked to indicate to what extent they agreed with the item using a 5-point scale (1 = strongly disagree, 5 = strongly agree). A higher score represents a higher level of conflict. We attained good reliability of internal consistency of scales. The coefficient α for this scale was α = 0.83.

#### Control variables

Previous studies have shown that gender and age correlate significantly with WFC (Grönlund, 2010; Powell and Greenhaus, 2010; Allen and Finkelstein, 2014). Therefore, we regarded gender and age as control variables when testing our hypotheses. Gender was dummy-coded (0 = female, 1 = male), and similarity in age was operationalized by the absolute difference score.

### Analysis method

This study used polynomial regressions and response surface analysis methods, which can provide more accurate results than other methods (Edwards and Parry, 1993). This method can overcome the limitations of the difference score method and the profile similarity index. In addition, response surface analysis presents a three-dimensional surface that can vividly depict two factors—the relationship between fit and misfit and how the relationship impacts WFC (Edwards and Shipp, 2007; Shanock et al., 2010).

According to the equation developed by Edwards and Parry (1993), this study constructed a measurement equation which is specified as follows:


$$\begin{array}{l} Z=b_{0}+b_{1}\left(X\right)+b_{2}\left(Y\right)+b_{3}\left(X^{2}\right)+b_{4}\left(X^{*}Y\right)+b_{5}\left(Y^{2}\right)+e \end{array}$$

This equation is used to measure both work demand-family resources and work resource-family demand. In this equitation, Z represents the outcome (WFC), X represents work demand (or work resource), Y represents family demand (or family resource), X^*^Y is the interactive term of corresponding factors, X^2^ and Y^2^ are squared factors; b_0_ represents intercept (constant term), b_1_ is the coefficient of X, b_2_ is the coefficient of Y, b_3_ is the coefficient of X^2^, b_4_ is the coefficient of X^*^Y, b_5_ is the coefficient of Y^2^, and e is random disturbance term.

The specific process we used for the analysis is as follows: we put two paired variables (X, Y), the squared terms (X2, Y2), and the interactive term (X^*^Y) into the regression equation specified in the analysis section above. First, we entered the control variables into the level 1 (M1); put the work demand (work resource) and family resource (family demand) into the level 2 (M2); put the squared terms of the work demand (work resource) and the family resource (family demand) and the interactive term in the level 3 (M3). In order to reduce multicollinearity and facilitate interpretation of the graphs, all predictor variables were scale-centered (Edwards and Parry, 1993). If the increment in R^2^ of model 3 (M3) is statistically significant, then the response surface analysis can be further performed. Finally, we plotted the three-dimensional response surface in which X and Y were plotted on the perpendicular horizontal axes, and Z was plotted on the vertical axis (Edwards and Parry, 1993).

In the three-dimensional response surface, along the “X = Y” line, we mainly determine how demand and resource fit impact WFC by calculating the slope of the surface a_1_, which is represented by (b_1_ + b_2_) and the curvature of the surface a_2_, which is represented by (b_3_ + b_4_ + b_5_) and the significance of slope and curvature; along with “X = –Y” line, we mainly determine how demand and resource misfit impact on WFC and the difference between fit and misfit by calculating the slope of the surface a_3_ which is represented by (b_1_-b_2_) and the curvature of the surface a_4_ which is represented (b_3_-b_4_+b_5_).

According to Podsakoff et al. (2003) and Bai et al. (2018), the conditions to test hypotheses related to the discrepancy between fit and misfit (H1 and H4) are (1) a_4_ is insignificant given that the curvature of the surface along this line does not differ from zero, the surface is flat along the X = –Y line, and (2) a_3_ is significant and positive, the dependent variable increases as X approaches Y from low to high (H1); a_3_ is significant and negative, the dependent variable decreases as X approaches Y from low to high (H4), dependent variable is at a medium level under these condition. The conditions to test hypotheses related to discrepancy of high and low demand-resource fit (H2 and H5) are (1) a_1_ is significant and positive, the dependent variable increases as the fit degree between X and Y increases (H2); a_1_ is significant and negative, the dependent variable decreases as the fit degree between X and Y increases (H5) and (2) a_2_ is insignificant given that the curvature of the surface along X = Y line does not differ from zero which means the surface was essentially flat along the X = Y line. The conditions to test hypotheses related to the discrepancy of two kinds of misfit (H3 and H6): (1) a_4_ is insignificant, the surface is essentially flat along the X = –Y line and (2) a_3_ is significant and positive, high X-low Y leads to higher WFC (H3); a_3_ is significant and negative, high X-low Y leads to lower WFC (H6).

## Results

### Common method biases test

To avoid common method bias, we selected mature scales verified by previous research and used the reversed score method in the same scales. In addition, we delivered questionnaires to participants from different organizations in different regions and emphasized the anonymity and confidentiality of the questionnaire to control the progress and ensure that collected feedback was reliable. Additionally, we handed out the survey of independent variables (work demand, family resource, work resource, and family demand) and dependent variables (WFC) in separate periods of time.

However, since all the research variables were answered by the same individuals, the common method biases may still exist. Therefore, we used the unmeasured latent method factor technique (Podsakoff et al., 2003). We introduced a method factor into the five-factor model in confirmatory factor analysis. The results showed that the five-factor model had the best result (shown in Table 1) (χ^2^ = 1008.007, df = 242, CFI = 0.904, IFI = 0.904, RMSEA = 0.069), but after introducing the method factor in the new model, the results showed that the model had not been set properly and could not be aggregated, indicating that the new model which included the method factor was unreasonable and thus common method biases were acceptable in this study.

**Table 1:** Compared results of confirmatory factor analysis.

**Model**	**χ^2^**	**df**	**χ^2^/df**	**RMSEA**	**CFI**	**GFI**	**IFI**
Five-factor model	1008.007	242	4.492	0.069	0.904	0.885	0.904
Three-factor model	3537.200	249	14.206	0.133	0.625	0.682	0.626
Single-factor model	6014.400	252	23.867	0.175	0.343	0.502	0.345

N = 745. The five-factor model includes work demand, family resource, work resource, family demand, and WFC; the three-factor model includes work demand and family demand which were combined into one factor, work resource and family resource which were combined into one factor and WFC; in the single-factor model, all five factors were combined into one factor.

### Confirmatory factor analysis

We used AMOS 19.0 to conduct confirmatory factor analysis on five variables (work demand, work resource, family demand, family resource, and WFC) to test the discriminant validity and compare the results among different models. Results are shown in Table 1. According to the results of confirmatory factor analysis, the five-factor model had the best result (χ^2^ = 1008.007, df = 242, CFI = 0.904, IFI = 0.904, RMSEA = 0.069) compared to the three-factor and single-factor model. The loadings of all the factors in the five-factor model were significant. In conclusion, the five-factor model is obviously better than other alternative models. These results meant that these five variables were empirically distinct from each other and represented five distinct constructs.

### Descriptive results

The means, standard deviations, and correlations for all measures are reported in Table 2. Results of descriptive statistics indicate that work demand is positively related to WFC; work resources and family resources are negatively related to WFC, and the relationship between family demand and WFC is insignificant.

**Table 2:** Means, standard deviations, correlations, and significance levels.

	**M**	**SD**	**1**	**2**	**3**	**4**	**5**	**6**	**7**
1. Gender	0.44	0.497	1						
2. Age	32.60	6.512	−0.123^**^	1					
3. Work demand	3.773	0.778	−0.142^**^	0.135^**^	1				
4. Family resource	3.786	0.735	0.150^**^	0.006	0.090^*^	1			
5. Work resource	3.333	0.765	0.020	−0.114^**^	0.074^*^	0.289^**^	1		
6. Family demand	3.373	0.923	0.192^**^	0.282^**^	0.093^*^	0.106^**^	0.127^**^	1	
7. WFC	3.115	0.796	−0.172^**^	0.138^**^	0.437^**^	−0.198^**^	−0.214^**^	−0.019	1

N = 745. ^*^p < 0.05, ^**^p < 0.01.

### Polynomial regressions and response surface analysis

#### Work demand and family resource

According to the analysis process and strategy demonstrated above, regression results of the relationship between work demand-family resource fit and misfit and WFC are depicted in Table 3. After taking squared terms (GX^2^ and JZ^2^) and the interactive term (GX^*^ JZ) into the regression model, R^2^ had a significant increment (ΔR2 = 0.03, *p* < 0.01). As a result, the model had a significant interpretation increment of impact on WFC, so response surface analysis can be conducted for further study.

**Table 3:** Polynomial regressions of work demand and family resource on WFC.

**Variables**	**WFC**		
	**M1**	**M2**	**M3**
Constant	−0.239	−0.258^*^	−0.267
**Control variables**			
Gender	−0.249^**^	−0.113^*^	−0.088
Age	0.014^**^	0.009^*^	0.008^*^
**Fit variables**			
WD (work demand)		0.424^**^	0.280^**^
FR (family resource)		−0.249^**^	−0.403^**^
WD^2^			0.098^**^
WD^*^ FR			0.061
FR^2^			0.084^**^
ΔR^2^		0.200^**^	0.030^**^
**Response surface features**			
**WD** = **FR Fit line**			
Slope (a_1_)			−0.12
Curvature (a_2_)			0.24^**^
**WD** = **–FR Fit line**			
Slope (a_3_)			0.68^**^
Curvature (a_4_)			0.12

N = 745, ^*^ p < 0.05, ^**^p < 0.01.

**Figure 1:**
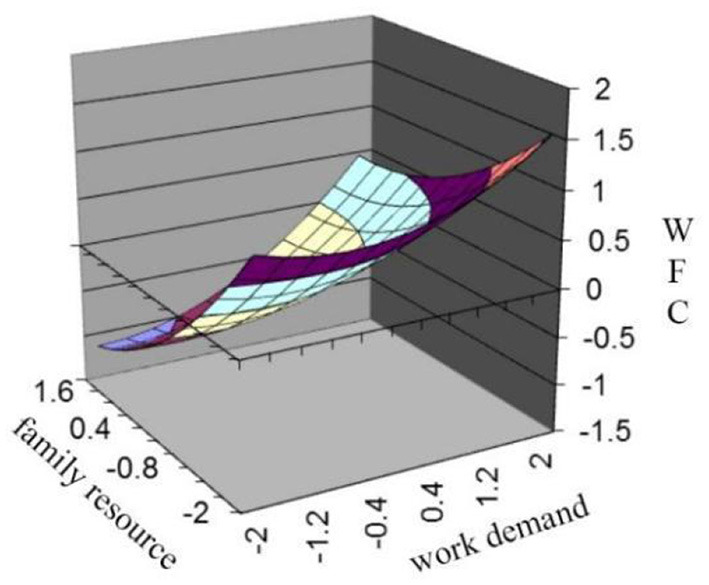
(Mis)fit effect of work demand and family resources on WFC.

Hypothesis 1 predicts that the fit between work demand and family resources is not the point at which an individual experiences the lowest WFC, and WFC will increase as work demand increases toward family resources and continues to increase as work demand exceeds family resources. Regression results and the slope and curvature along the fit line and misfit line, which are calculated by equations conducted by Edwards and Parry (1993), are shown in Table 3. Statistical results indicate the curvature of the misfit line is statistically insignificant (a4 = 0.12, n.s.), but the slope is statistically significant and positive (a3 = 0.68, *p* < 0.01), which indicates that the surface along the misfit line is upward from left to right. In other words, WFC increases when work demand increases toward family resources, and when work demand exceeds resources, WFC still increases. To facilitate the interpretation of the results, a graph of the surface plot is depicted in Figure 1. According to the figure, the surface along the misfit line is essentially a slanted and flat plane, while along the misfit line (from the left corner to the right corner), WFC gradually increased. The effect of the misfit or along the misfit line is solely presented in **Figure 3**. The figure also verifies that WFC increases in the process, and the paired factors gradually change from low work demand-high family resource to high work demand-low family resource. When work demand is proximal to family resources, WFC is at the medium level. These results provide evidence for Hypothesis 1.

**Figure 2:**
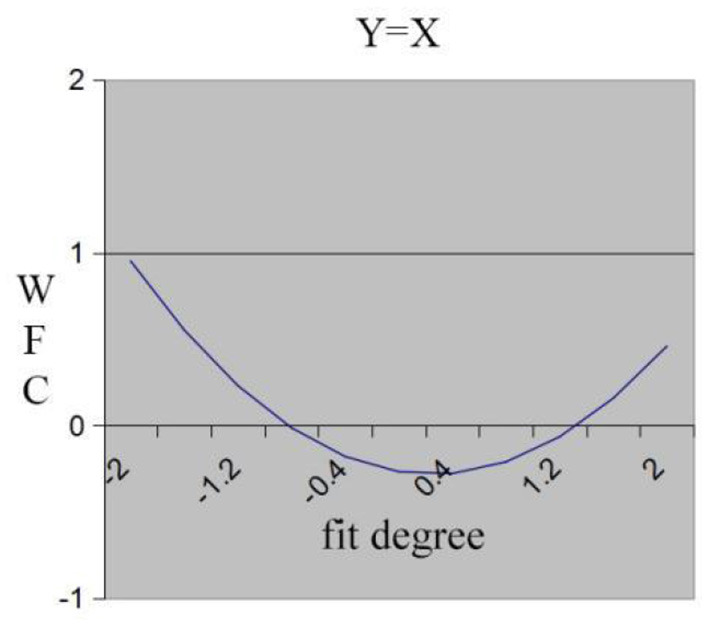
Fit line effect of work demand and family resource on WFC.

Hypothesis 2 predicts that individuals who experience low work demand-low family resource fit will perceive high WFC compared with those who experience high work demand-high family resource fit. We assumed that high-high fit leads to higher WFC compared to low-low fit. However, statistical results shown in Table 3 indicate that the slope is not statistically significant (a_1_ = −0.12, n.s.), but the curvature of the fit line is statistically significant and positive (a_2_ = 0.24, *p* < 0.01), which means that the surface along the fit line was concave. There was a U-shaped relationship between work demand-family resource fit and WFC. The fit line projected onto the response surface is shown in Figure 2, which also presents the same result. In Figure 2, the x-axis shows the degree of fit between work demand (X) and family resources (Y), with the degree of fit increasing from left to right. In other words, WFC decreases at first and increases along the line of fit. Therefore, these results partially support Hypothesis 2: under a certain condition, the WFC of the high-high fit is greater than the low-low fit.

**Figure 3:**
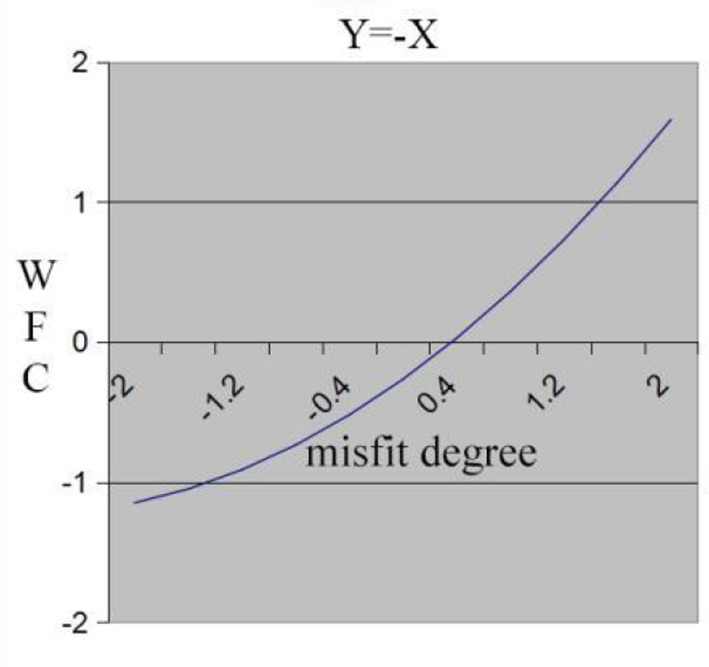
Misfit line effect of work demand and family resource on WFC.

Hypothesis 3 predicts that individuals who experience high work demand-low family resource misfit (“inferior” misfit) will perceive higher WFC compared to those who experience low work demand-high family resource misfit (“superior” misfit). We tested the hypothesis in the same fashion as testing H1 and used the same results in Table 3. Insignificant curvature of the misfit line, as well as significant and positive slope, indicate that WFC decreases along the misfit line from demand exceeding resource (X>Y region) to resource exceeding demand (X < Y region). The surface in Figure 1 slopes upward from left to right, so the high demand-low resource misfit (“inferior” misfit) leads to the highest WFC. Figure 3 demonstrates the same results. In Figure 3, the x-axis shows the degree of misfit between work demand (X) and family resource (Y), with a “superior” misfit gradually turning into an “inferior” misfit from left to right. Hypothesis 3 is verified.

#### Work resource and family demand

As the analysis process and strategy demonstrated above, regression results of the relationship between work resource-family demand (mis)fit and WFC are depicted in Table 4. After taking squared terms (GZ^2^ and JX^2^) and the interactive term (GZ^*^ JX) into the regression model, R^2^ had a significant increment (ΔR^2^= 0.019, *p* < 0.01), so response surface analysis can be conducted for further study.

**Table 4:** Polynomial regressions of work family and demand resource on WFC.

**Variables**	**WFC**		
	**M1**	**M2**	**M3**
Constant	0.239	−0.085	0.059
**Control variables**			
Gender	−0.249^**^	−0.251^**^	−0.251^**^
Age	0.014^**^	0.012^**^	0.009^**^
**Fit variables**			
WR (work resource)		−0.211^**^	−0.202^**^
FD (family demand)		0.010	0.015
WR^2^			0.072^**^
WR^*^ FD			−0.059
FD^2^			−0.098^**^
ΔR^2^		0.040^**^	0.019^**^
**Response surface features**			
**WR** = **FD Fit line**			
Slope (a_1_)			−0.19^**^
Curvature (a_2_)			−0.08
**WR** = **–FD Fit line**			
Slope (a_3_)			−0.22^**^
Curvature (a_4_)			0.03

N = 745, ^**^p < 0.01.

**Figure 4:**
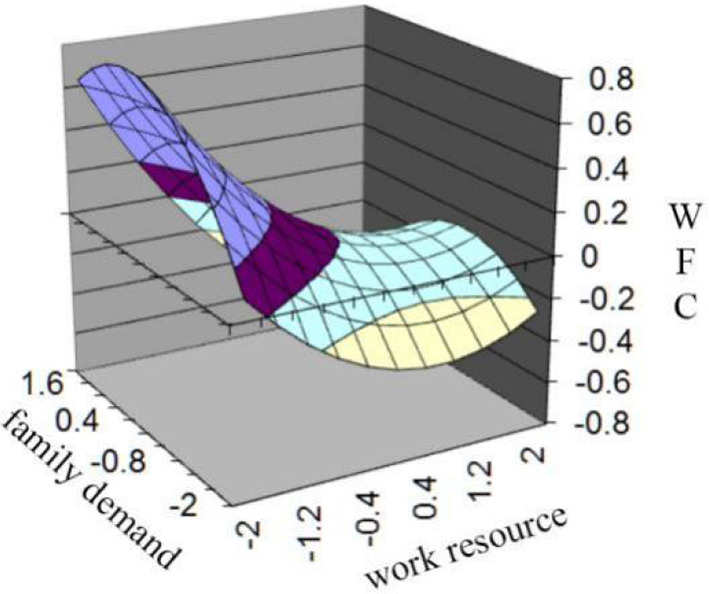
(Mis)fit shows the effect of work resources and family demand on WFC.

Hypothesis 4 predicts that the fit between work resource and family demand is not the best point at which an individual experiences the lowest WFC, and WFC will decrease as work resource increases toward family demand and continues to decrease as work resource exceeds family demand. Regression results and the slope and curvature along the fit line and misfit line are shown in Table 4. Results show that the curvature of the misfit is insignificant, but the slope is significant (a4 = 0.03, n.s.; a3 = −0.22, *p* < 0.01), indicating that the surface along the misfit line is essentially a slanted and flat plane. Along the misfit line, WFC gradually decreases when work resource approaches family demand and still decreases as work resource exceeds family demand. To facilitate the interpretation of the results, a graph of the surface plot is depicted in Figure 4. Along the misfit line, WFC decreases as work resources increase toward family demand and continues to decrease as work resources exceed family demand; WFC is at the medium level when two paired factors fit with each other. All these results support Hypothesis 4.

**Figure 5:**
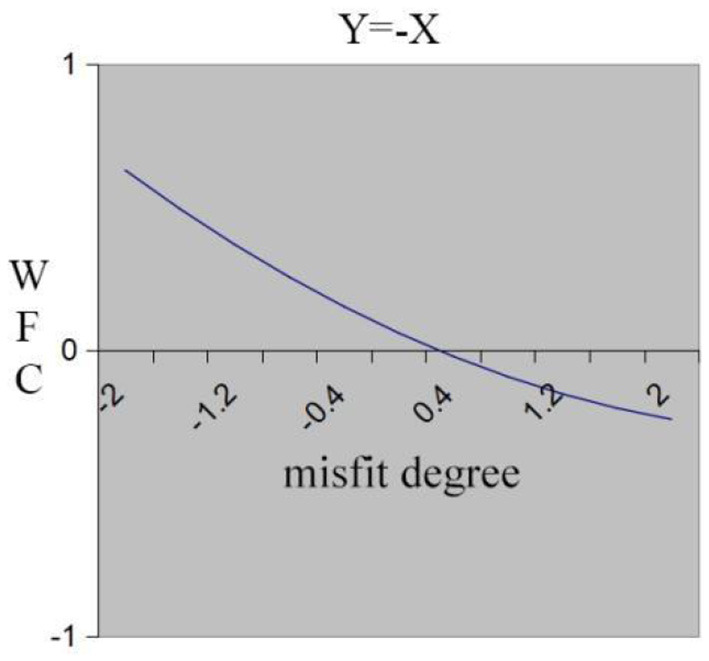
Misfit line effect of work resource and family demand on WFC.

The effect of misfit or along the misfit line is solely presented in Figure 5. In Figure 5 the x-axis shows the degree of misfit between work resource (X) and family demand (Y), with an “inferior” misfit gradually turning into a “superior” misfit from left to right. The figure also verifies that WFC decreases in the process, and the paired factors gradually change from low work resource-high family demand misfit to high work resource -low family demand misfit. WFC is at the medium level when work demand is proximal to family resources. Hypothesis 6 predicts that individuals who experience low work resource-high family demand misfit (“inferior” misfit) will perceive higher WFC compared to those who experience high-low misfit (“superior” misfit). Given that the slope is significantly negative along the misfit line, and the surface essentially slopes downward, WFC increases from the “inferior” misfit region to the “superior” misfit region. Results support Hypothesis 6.

**Figure 6:**
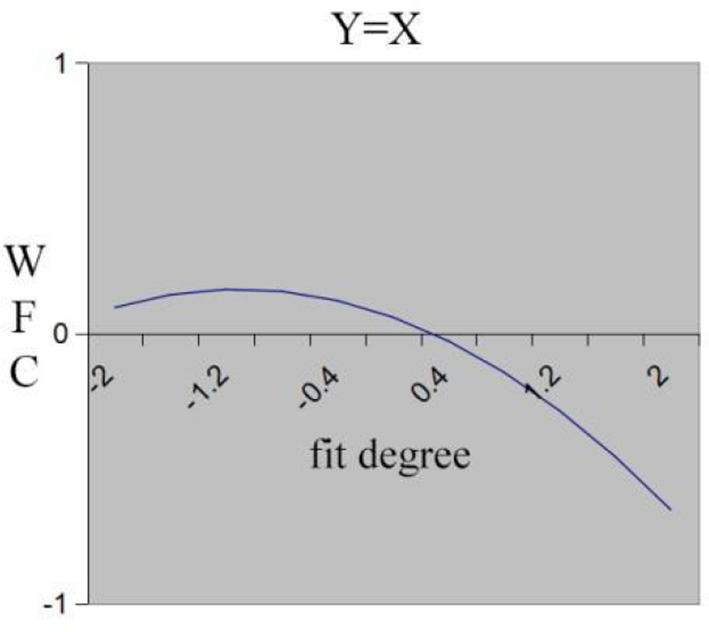
Fit line effect of work resource and family demand on WFC.

Hypothesis 5 predicts that individuals who experience low-low fit will perceive higher WFC compared to those who experience high-high fit. The curvature of the fit line is insignificant (a_2_ = −0.08, n.s.), and the slope is significantly negative (a_1_ = −0.19, *p* < 0.01). As is shown in Figure 4, along the fit line, the surface slopes downward. Figure 6 also shows that WFC decreases as work resources and family demand increase simultaneously. In Figure 6, the x-axis shows the degree of fit between work resources (X) and family demand (Y), with the degree of fit increasing from left to right. High work resource-high family demand fit reaches lower WFC compared to low work resource-low family demand fit. These results provide evidence for Hypothesis 5.

## Discussion

We used the P-E theory and JD-R model to research the effects of fit and misfit between demand and resource on WFC through polynomial regression and response surface analysis.

### The relationship between work demand and family resource (mis)fit and WFC

It was found that sufficient family resources and low work demands can effectively reduce the individuals' perceived WFC. As depicted in Figure 1, we can see that when the work demand is much higher than the family resource, an individual will experience the highest WFC. When the work demand and the family resources are aligned, the perceived WFC is at a medium level.

Chinese always maintain the tradition of “job priority.” Issues in the work domain (work overtime, performance decline) are more likely to impact individuals' behavior and attitude. Different from Western cultures, we consider work as a means to enhance family welfare and support the whole family instead of competing with family; thus, we work to live (Aryee et al., 1999; Wang et al., 2004). We spend more time and energy on work and usually get support from family members. We focus on fulfilling work responsibilities and use family resources to meet work demands. For example, an employee tends to work overtime to finish work instead of taking part in a family reunion. Thus, family resources are easily transferred into the work domain to meet work demands.

The positive effects of family resources can offset the negative effects of work demand. “Inferior” misfit occurs when the resources are less than the demands. Under this condition, individuals possess insufficient family resources and high work demands, and they must devote more time and energy to work and have no time or energy to take care of their family affairs. Therefore, they feel extremely high WFC. A “superior” misfit occurs when the resources are higher than the demands. Sufficient family resources enhance the individual's ability to cope with work issues. They effectively meet the work demands, and the family will not be interfered with by work issues. For an employee who has flexible work hours in the workplace with family members who can share household duties, WFC is hardly ever a problem.

The results also indicate that the fit of the paired factors has a U-shaped effect on WFC. Different from our prediction, the low degree of fit also leads to high WFC. As described in the COR (Hobfoll, 2001) theory, there is a loss of spiral effect in resources. Every job has its own basic demands, and the Chinese are inclined to use family resources to meet the job demands. When individuals consume family resources, low family resources will spiral loss at an accelerated rate. As a result, individuals are more susceptible to work demands and will be more likely to experience high WFC. Our results also demonstrate that family resources have a significantly negative impact on WFC, so family resources also play an important role in the fit effect. In extreme situations, the low-low fit is a kind of “compromised” fit that scales down the expectations in work and family domains. Therefore, when work demand and family resources are simultaneously high and low, individuals will perceive high WFC.

### The relationship between work resource and family demand (mis)fit and WFC

The results of the study show that compared to “inferior” misfit, “superior” misfit leads to low WFC. Sufficient work resources mean that individuals have more ability to meet family demands and perceive low WFC. In addition, compared to low work resource-family demand fit, high work resource-family demand fit leads the individual to experience low WFC. As we illustrated above, the Chinese consider jobs as a method to improve family welfare, and resources from work are used to meet family demands. Work resources are easily transferred into the family domain to meet family demand. The internal and external rewards of the work improve the individual's ability to cope with family affairs and meet the material and spiritual demands of the family, which leads to low perceived WFC. In China, family demand is not an obstacle in most cases as Chinese tend to put more time and energy into work to gain more resources to enhance family welfare.

## Implications

### Theoretical implications

Research on the antecedents associated with WFC has been extensive and rich, but there is still an imbalance between research on factors in family and work domains and a relatively homogeneous study of variables. The theory of person-environment fit enlightens us that in the study of WFC factors, no single factor operates independently, as there exist mutual influences between them. The interaction of factors in family and work domains will influence an individual's conception of WFC. It has been shown that demands and resources in both work and family domains, including work intensity, leadership support, child care, and family support, influence WFC. Based on the foundation of previous research, this study proposed a multi-factorial interaction and verified the different effects of fit and misfit, different degrees of fit, and different directions of misfit of the factors on WFC, which broadened the study of the mechanisms that trigger WFC. Specific theoretical implications include the following:

First, most of the studies that have been carried out, although relatively abundant, have considered WFC only in a unilateral, namely work-related or family-related context. These studies focused more on the work side, ignoring the impact of family on work. For example, family resources complement work demands, and family demands interfere with work resources. This study not only considered the impact of resources and demands in the work field on WFC but also included non-negligible resources and demands in the family field into the research, enriching the unilateral perspective of previous research on the mechanisms that trigger WFC.

Second, there is relatively limited research on the interaction of multiple factors on WFC, and the research focus is only on the question of whether there is a fit, defining the situation where resources meeting demands as fit, and the more resources exceeding the demands, the higher the level of fit. Misfit occurs when demands exceed resources, and as the level of demands exceeding resources increases, the level of misfit also increases. This definition of fit and misfit is too simplistic and overlooks the dynamic process of fit and misfit. We studied the comprehensive dynamic process of fit and misfit instead of only fit in previous studies. Resources are considered more positive than demands, and the interaction of resources and demands is more complex than a single factor. This study comprehensively considered three situations where resources are less than, equal to, and greater than demands, ad made up for existing studies that solely consider the condition of fit or misfit.

Third, we applied polynomial regression and response surface analysis to the work-family domain fit research. The dynamic development process of fit and misfit is clearly demonstrated through 3D images. Compared with the previous research fit method, the polynomial regression and response surface analysis method make up for the defects, such as the reduction of measurement reliability and the confusion between the effects of demands and resources. This method can verify the three aspects of hypotheses, including the discrepancy between fit and misfit, the discrepancy of the different degrees of fit, and the discrepancy of the different degrees of misfit, while ensuring a clear theoretical concept and reliability.

### Practical implications

The negative effects of WFC cannot be ignored. It can lead to job burnout, higher turnover, increased absenteeism, poorer performance, even stress, depression, and suicide (Frone et al., 1992; Carlson et al., 2000). Therefore, employees and employers should take measures to reduce or even eliminate WFC perceived by employees.

As a result of dramatic changes in work and family domains in recent years, demands and resources in work and family impact WFC in different ways and to different degrees. Only focusing on a single factor is not enough. Employers and employees have more flexibility as they can change any of these factors to achieve the best combination, especially when a factor is difficult to adjust.

Employers should help employees achieve “superior” misfit by reducing work demands and increasing work resources. First, reduce work demand. Companies should rationally design job descriptions to eliminate ambiguous job responsibilities and arrange reasonable work schedules to help employees accommodate family responsibilities. Second, increase work resources. Work demand is always difficult to reduce because of the rigid requirements of any job. For example, it is well believed that a sales manager has business trips often. In this condition, companies need to provide sufficient work resources (e.g., dependent care benefits) to ensure that employees experience low WFC when family demands are high. A method is to implement a flexible work system reasonably, which includes flexible work hours, flexible work content, flexible workplace, and flexible holidays (Li and Liu, 2014). Flexible work hours mean employees can arrange work hours flexibly and autonomously under the premise of ensuring the completion of enough working hours. For example, employees who have urgent family affairs can leave in advance. Flexible work content means employees can arrange the work content flexibly and autonomously under the premise of ensuring the completion of the requested work content. For example, employees can shift work tasks to other colleagues. A flexible workplace means that the workplace can be self-selected under the premise of ensuring work efficiency so that employees can choose to work at home to better fulfill their family obligations. Flexible holidays allow employees to take time off on workdays to handle personal matters, increase parental leave for sick children, and take time off to take care of the elderly.

In addition, assistance programs can also increase their work resources: (1) professional training and guidance for employees and their families to deal with work and family affairs, such as time management skills to rationally arrange work and family time, effective communication methods to improve relationships with family members and job-related skills and (2) providing and designing systematic, long-term employee assistance and welfare programs including child care centers, home service information disclosure and appropriate family allowance (education fund, medical assistance, etc.).

Demands are usually difficult to change, and the cost of adding work resources to the companies will be extremely high. Many companies have insufficient time and energy to support adding resources. Employees should take the initiative to achieve a “superior” misfit. First, lower the expectations of work. Employees can actively give up overtime for extra payment and voluntarily give up higher positions, which demand devoted time and energy. Second, increase work resources. Employees can proactively build good relationships with superiors and colleagues to get their help. The sense of accomplishment and self-realization achieved from work is also a way to increase work resources. Third, reduce family demands. Employees can reduce household expenses and family expectations, etc. Finally, increase family resources. Employees can actively seek instrumental support in the family domain, such as sharing housework with spouses, getting help from parents to take care of children, and hiring babysitters to augment family resources. In addition, having different career development phases with the spouse is also a strategy to avoid busy times of work; that is, one spouse chooses to give up promotion to take care of the family while the other is in the career promotion period. Emotional support from the family, in the form of encouragement, compliments, and comfort is also an important resource.

In extreme situations such as the COVID-19 pandemic, both work demands and family demands tend to increase, while the corresponding resources from work and family may decrease. For example, the discontinuity in business production and operations caused by the COVID-19 pandemic required employees to cooperate with the company, thereby increasing the uncertainty of working hours. Moreover, the suspension of business operations during the COVID-19 pandemic could lead to a significant wage cut. During this period, family responsibilities increased when more attention to the health conditions of older adult family members and children was required. As a result, the pandemic exacerbated the level of misfit, leading to higher levels of WFC. Therefore, in similar extreme conditions, we can proactively lower our work expectations and maintain open and honest communication with superiors, colleagues, and family members to seek emotional support to reduce WFC.

## Limitations and future research

This study has some limitations. First, the use of self-reported data increased the common method biases. Even though data was obtained by measuring factors separately, the factors were still reported by the same person. Future research could collect data at multiple time points to overcome this problem.

Second, the work-family balance has two effects: conflict and facilitation, which coexist in one's life to influence an individual's feelings. Moreover, work and family influence each other in two directions: work-to-family and family-to-work. This study started in the work domain and only studied the impacts of work-to-family conflict. Researchers can extend future research to the other effects and direction of work-family balance and conduct comprehensive studies on the effects of fit and fit between resources and demands imposed on the overall work-family balance mechanism.

Third, this study focused on the impact of the dynamic process of fit and misfit of work demands and family resources, as well as of work resources and family demands on WFC. There may be certain mediating and moderating variables in the process, which can be further investigated in depth in subsequent studies.

Fourth, this study only investigated the dynamic relationships between demands and resources in Chinese culture, and these dynamic relationships may show different characteristics in different cultures, which may lead to different impacts of the combined and interactive relationships between demands and resources on WFC. Therefore, it is of certain academic and practical value to study the dynamic relationships between demands and resources in different cultures in future research.

## Data availability statement

The original contributions presented in the study are included in the article/Supplementary material, further inquiries can be directed to the corresponding author/s.

## Ethics statement

The requirement of ethical approval was waived by the School of Economics and Management, Yanshan University for this study involving human participants because it did not involve any human clinical experiments or animal experiments, and the anonymous method was used to protect the privacy of participants. The studies were conducted in accordance with the local legislation and institutional requirements. The participants provided their written informed consent to participate in this study.

## Author contributions

TL: Writing – original draft. ZH: Supervision, Writing – review & editing. LH: Supervision, Writing – review & editing. MM: Conceptualization, Formal analysis, Investigation, Methodology, Resources, Validation, Writing – review & editing.
